# Extensive host-switching of avian feather lice following the Cretaceous-Paleogene mass extinction event

**DOI:** 10.1038/s42003-019-0689-7

**Published:** 2019-11-29

**Authors:** Robert S. de Moya, Julie M. Allen, Andrew D. Sweet, Kimberly K. O. Walden, Ricardo L. Palma, Vincent S. Smith, Stephen L. Cameron, Michel P. Valim, Terry D. Galloway, Jason D. Weckstein, Kevin P. Johnson

**Affiliations:** 1 Illinois Natural History Survey, Prairie Research Institute, University of Illinois, Champaign, IL USA; 2 Department of Entomology, University of Illinois, Urbana, IL USA; 3 Department of Biology, University of Nevada, Reno, NV USA; 4 Department of Entomology, Purdue University, West Lafayette, IN USA; 5 Museum of New Zealand Te Papa Tongarewa, Wellington, New Zealand; 6 Department of Life Sciences, The Natural History Museum, London, UK; 7 Biotério da Universidade Iguaçu, Nova Iguaçu, RJ Brazil; 8 Department of Entomology, University of Manitoba, Winnipeg, Manitoba Canada; 9 Department of Ornithology, Academy of Natural Sciences of Drexel University, Philadelphia, PA USA

**Keywords:** Coevolution, Phylogenetics

## Abstract

Nearly all lineages of birds host parasitic feather lice. Based on recent phylogenomic studies, the three major lineages of modern birds diverged from each other before the Cretaceous-Paleogene (K-Pg) mass extinction event. In contrast, studies of the phylogeny of feather lice on birds, indicate that these parasites diversified largely after this event. However, these studies were unable to reconstruct the ancestral avian host lineage for feather lice. Here we use genome sequences of a broad diversity of lice to reconstruct a phylogeny based on 1,075 genes. By comparing this louse evolutionary tree to the avian host tree, we show that feather lice began diversifying on the common ancestor of waterfowl and landfowl, then radiated onto other avian lineages by extensive host-switching. Dating analyses and cophylogenetic comparisons revealed that two of three lineages of birds that diverged before the K-Pg boundary acquired their feather lice after this event via host-switching.

## Abstract (web summary)

Robert de Moya et al. use comparative genomics of avian lice to reconstruct the phylogeny of these parasites. They show that feather lice diversified on the common ancestor of waterfowl and landfowl, and then radiated onto other avian lineages through host-switching.

## Introduction

The mass extinction event at the Cretaceous–Paleogene (K-Pg) boundary, 66 million years ago, had a major impact on the Earth’s biota, including extinction of the dinosaurs. Only three lineages of extant birds (Palaeognathae, Galloanserae, and Neoaves) survived this event^1,2^. The Palaeognathae includes the flightless ratites (ostriches, rheas, kiwis, cassowaries, and emus) and tinamous, which diverged from all other extant avian lineages between 72 and 102 Mya^1,2^. The Galloanserae includes waterfowl (Anseriformes) and landfowl (Galliformes), which diverged from the remaining avian lineages (Neoaves) between 71 and 90 Mya^1,2^. All three of these groups of birds host ectoparasitic feather lice^3^, but it is currently unclear on which avian ancestor these parasites originated.

Feather lice (Philopteridae), with more than 3000 described species^3^, comprise the most diverse family of parasitic lice (Phthiraptera). They have intimate relationships with their avian hosts and spend their entire lifecycle on the body of the host, consuming downy feathers^3^. A recent phylogenomic study suggested that the radiation of feather lice occurred following the K-Pg boundary^4^. This result implies that only one lineage of feather lice passed through the K-Pg mass extinction and that the presence of feather lice on all three extant bird lineages is a result of host-switching. However, that study did not extensively sample feather lice from Palaeognathae, except for feather lice from the tinamous^4^. Thus, a comprehensive phylogeny of feather lice, including lice from all orders of ratites is needed to identify the ancestral host of feather lice and understand the origins of this group of parasites.

We conducted a cophylogenomic analysis of feather lice and birds to reconstruct the ancestral host of these parasites and uncover the evolutionary origins of feather lice. We included feather louse samples from all orders of Palaeognathae, as well as from a wide diversity of other birds for a total of 60 feather louse species. This taxonomic sampling of feather lice included lice from nearly all avian orders and was selected to compile a comparable data set to recent avian phylogenomic studies^1,2^ making cophylogenetic comparisons at this phylogenetic scale possible. We constructed a phylogenomic data set consisting of 1075 genes from whole-genome sequencing reads to reconstruct a feather louse phylogeny and the timing of diversification of this group of parasites. We compared the reconstructed evolutionary tree of feather lice with two major avian host phylogenies that were also derived from phylogenomic data sets^1,2^. These two avian phylogenies are similar in many respects, including the same topological relationships among Palaeognathae, Galloanserae, and Neoaves. They differ primarily in some rearrangements among groups within Neoaves. Thus, our cophylogenetic analyses incorporate this uncertainty in the avian tree.

## Results

### Phylogenomic and dating analyses

**Fig. 1:**
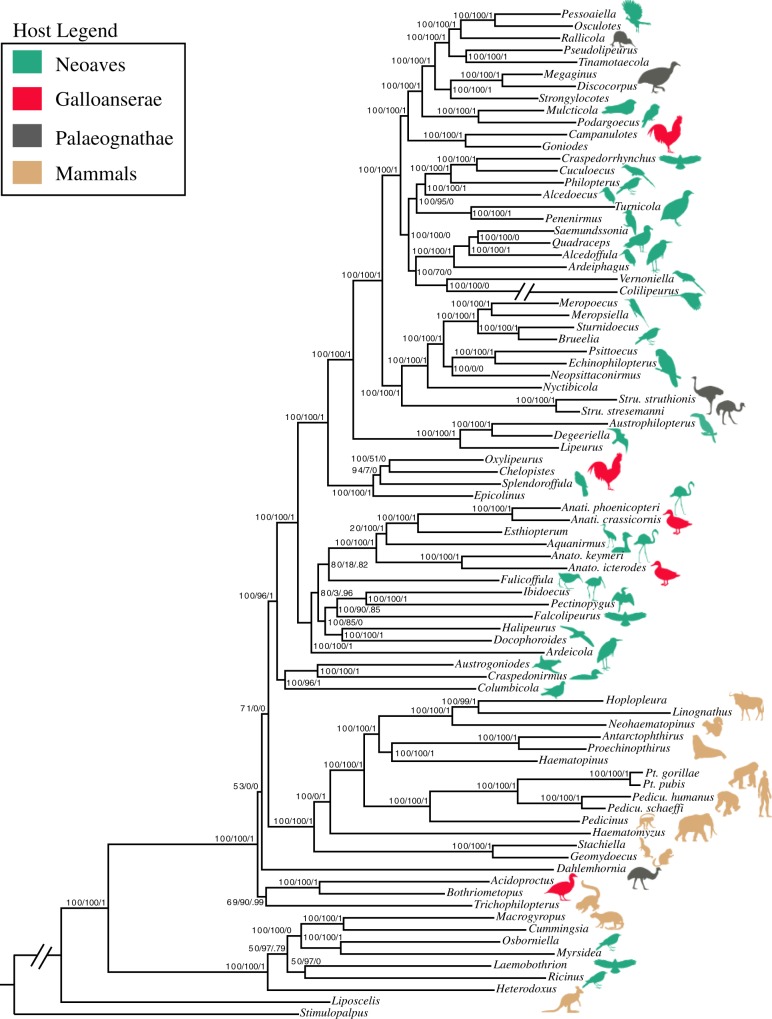
Phylogeny of feather lice and outgroups based on a partitioned maximum likelihood search with 3rd codon positions removed. Summary support for other analyses are provided on nodes. The first value is the bootstrap support from the maximum likelihood analysis of the data with 3rd positions removed. The second value is bootstrap support based on maximum likelihood analysis of all nucleotide sites. The third value is the local posterior probability from the Astral gene tree analysis. Note: Two exceptionally long branches are broken for the purposes of graphical display. Images are obtained from phylopic.org (Cuculidae: Lip Kee Yap; Struthio: Matt Martyniuk and T. Michael Keesey; Rhea: Darren Naish and T. Michael Keesey; Falco: Liftarn; Tauraco: Lisa M. “Pixxl”, John E. McCormack, Michael G. Harvey, Brant C. Faircloth, Nicholas G. Crawford, Travis C. Glenn, Robb T. Brumfield and T. Michael Keesey; Podiceps: Doug Backlund, John E. McCormack, Michael G. Harvey, Brant C. Faircloth, Nicholas G. Crawford, Travis C. Glenn, Robb T. Brumfield and T. Michael Keesey; Phalacrocoracidae: L. Shymal; Fulmarus: Bennet McComish and Avenue; Aptenodytes: Neil Kelley; Columbidae: Dori and Nevit Delmin; Sciurus: Anthony Caravaggi; Cavioidea: Zimices; Macropodiformes: T. Michael Keesey and Tony Hisgett; all images are modified under license: https://creativecommons.org/licenses/by-sa/3.0/legalcode) (Hoatzin: Warren H. and T. Michael Keesey; Tinamus: Darren Naish and T. Michael Keesey; Buteo: Shyamal; Laridae: Rebecca Groom; Ardea: Rebecca Groom; Coliidae: Joseph Wetsy, John E. McCormack, Michael G. Harvey, Brant C. Faircloth, Nicholas G. Crawford, Travis C. Glenn, Robb T. Brumfield and T. Michael Keesey; Gavia: John E. McCormack, Michael G. Harvey, Brant C. Faircloth, Nicholas G. Crawford, Travis C. Glenn, Robb T. Brumfield and T. Michael Keesey; Connochaetes: Jan A. Venter, Herbert H. T. Prins, David A. Balfour, Rob Slotow and T. Michael Keesey; Pan: T. Michael Keesey and Tony Hisgett; Dromaius: Darren Naish and T. Michael Keesey; all images are modified under license: https://creativecommons.org/licenses/by/3.0/legalcode).

The relationships among major groups of lice reconstructed by our phylogenomic analyses are similar to previously published results^4^, and most nodes (>80%) received 100% bootstrap support. The monophyly of feather lice is supported (Supplementary Fig. 1) when all nucleotide sites in the alignment are analyzed. However, when third codon positions are excluded from the analysis, a group of mammal lice is embedded within some of the earliest diverging feather louse lineages (Fig. 1). We conducted cophylogenetic analyses using all combinations of avian and louse phylogenies, to account for these phylogenetic uncertainties.

**Fig. 2:**
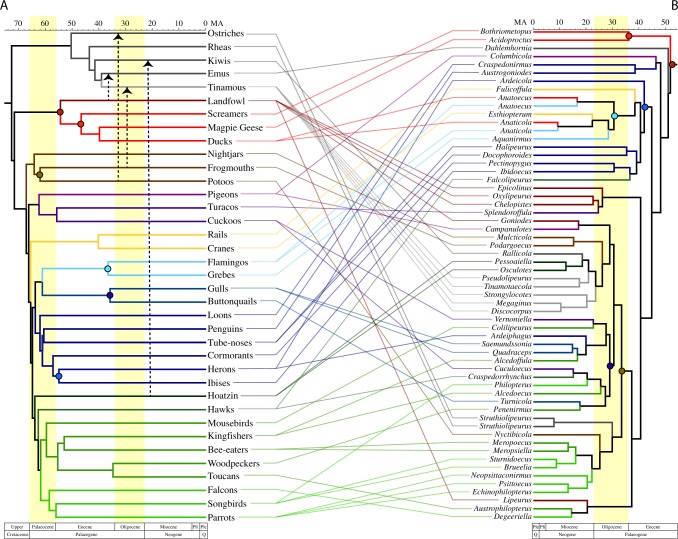
Cophylogenetic comparison of feather lice (**b**) with their avian hosts (**a**). Colors of branches in the feather louse phylogeny corresponds to the colors of the branch of major groups of their avian hosts. The feather louse tree is based upon the partitioned maximum likelihood analysis of the concatenated matrix with 3rd codon positions removed (Fig. 1). Timescale corresponds to results from dating analyses of the feather louse tree performed in MCMCtree. The bird host tree is based on Prum et al.^2^ Dashed arrows indicate reconstructed host-switches from other avian groups to Palaeognathae as indicated by the Jane cophylogenetic analyses. Colored circles identify cospeciation events between birds and feather lice as indicated by the Jane analysis.

Using the louse phylogenomic data set, we also conducted a dating analysis using calibration points external to the clade of feather lice. These dating analyses indicate that feather lice began radiating around 50 Mya, somewhat after the K-Pg boundary (Fig. 2), which is similar to previously published studies^4^. Thus, feather lice began to diversify following the origin of most modern avian orders^1,2^.

### Cophylogenomic analyses between feather lice and avian hosts

Although comparisons of the louse parasite tree to the avian host tree (Fig. 2) using distance-based methods (Parafit^5^ and PACo^6^) revealed significant congruence (*P* < 0.05) between the two tree topologies, the number of reconstructed codivergence events was relatively small (6 or 7 depending on comparison). Rather, overall the cophylogenetic analyses suggested that multiple host-switches have taken place by lice among modern groups of birds. The results of Jane^7^ cophylogenetic analyses indicated that the ancestral host of feather lice was the common ancestor of the Galloanserae (waterfowl and landfowl), and this result was stable across all possible combinations of host and parasite trees. The estimated timing of the earliest codivergence between Galloanserae and their feather lice (maroon circle in Fig. 2) is also similar (50 Mya for feather lice and 55 Mya for the Galloanserae in the Prum et al. tree^2^). This timing implies that neither the common ancestor of Palaeognathae nor Neoaves hosted feather lice (or at least not one from an extant lineage).

The fact that the earliest divergence in feather lice is associated with the divergence between waterfowl (Anseriformes—screamers, magpie geese, and ducks) and landfowl (Galliformes) indicates that Palaeognathae and Neoaves must have acquired their feather lice via host-switching. Cophylogenetic analyses suggest host-switching occurred from other birds to palaeognaths at least three times (Supplementary Tables 1 and 2, Supplementary Fig. 2) depending on the host tree evaluated (three host-switches in comparison with the Jarvis et al. tree^1^ and four host-switches in comparison with the Prum et al. tree^2^). All analyses indicate one of these host-switches to Palaeognathae was from an ancestor within Galloanserae to the ancestor of emus. Furthermore, two consistent host-switches to palaeognaths originated from the ancestors of frogmouths and potoos, two early diverging lineages of Neoaves.

Among the feather lice of Neoaves, some intriguing cophylogenetic patterns emerge. For example, both of the recent avian phylogenomic studies recovered a large group (Aequorlitornithes) of water associated birds^1,2^. We also found that the feather lice of these birds tended to be closely related. However, feather lice from some other birds associated with water, such as ducks and cranes, also fall within this clade of lice. Cophylogenetic reconstruction (Supplementary Fig. 2) suggests that ducks and cranes acquired their feather lice through host-switching from an ancestral flamingo^8^ (a member of the Aequorlitornithes). Thus, host-switching in these cases might have been facilitated by a shared aquatic habitat. We also reconstructed the acquisition of feather lice from two main lineages of predatory birds, hawks and falcons, as being the result of host-switching from other avian lineages (Supplementary Fig. 2). Hawks, for example, sometimes acquire feather lice from their prey^9^. Thus, predation on other birds may facilitate host-switching by feather lice to raptors^10^.

## Discussion

The pattern of feather louse diversification is characterized by extensive host-switching rather than codivergence. Complex host-switching patterns of feather lice among bird hosts may be explained by observations of the dispersal behavior of extant feather louse species. Numerous accounts have documented the ability of feather lice to attach to winged hippoboscid flies (louse flies, Diptera) and disperse between hosts via phoretic hitch-hiking^11,12^. The divergence of avian feeding hippoboscid flies is estimated to have occurred up to around 52 Mya^13^ providing opportunities for ancient host-switching via phoresis. Other opportunities for host-switching may also exist, such as sharing of nest sites or dust baths, which may allow for host-switching among distantly related groups of birds through indirect contact^14^. Host-switching may also be easier for feather lice compared with other groups of parasitic lice, because feather lice do not trigger a host immune response when feeding on downy feathers^15^. For example, blood-sucking lice (Anoplura) elicit an immune response when feeding on the blood of their mammalian hosts^16^. This may limit the ability of blood-feeding lice to successfully switch hosts in comparison with feather-feeding lice, which encounter no immune response from a novel host^15^. This ability to more easily switch among avian hosts may have contributed to the diversification of feather lice, which would help to explain why the diversity of feather lice exceeds the total species diversity of all other groups of parasitic lice combined^3,17^.

This study is one of the most comprehensive cophylogenetic studies of parasites across extant avian diversity. Host-switching has proven to be a relatively common phenomenon across lineages associated with birds. For example, the pattern of host-switching among major avian host lineages is similar to patterns seen on smaller cophylogenetic scales between feather mites and their avian hosts^18–20^. Extensive host-switching has been inferred in this mite system, which has a similar transmission ecology^18,19^ to that of lice, despite ecological specialization^20^. Extensive host-switching has also been detected in the malaria endoparasites that infect birds^21^. The mobile nature of bird species likely promotes host-switching of parasites given similar patterns of host-switching between internal and external parasites.

In summary, both the timing and phylogenetic pattern of feather louse diversification indicates that the birds in the groups Palaeognathae and Neoaves did not inherit their feather lice from a common ancestor. Rather they acquired them several times independently from other lineages of birds through host-switching. The fact that palaeognaths diverged from other birds well before the K-Pg boundary, while feather lice diversified only after this mass extinction event, supports this hypothesis. Instead, feather lice began diversifying on the common ancestor of waterfowl and landfowl (Galloanserae) and radiated across birds through extensive host-switching.

## Methods

### Taxonomic sampling

To evaluate the phylogenetic position of the feather lice of ratites and tinamous (Palaeognathae), we sequenced the genomes of 60 species of feather lice and 24 outgroup species including other groups of chewing and sucking lice from birds and mammals and two free-living bark lice. This sampling included data from 46 previously published genomic data sets, as well as 38 newly sequenced genomes^4^ (Supplementary Table 3). Our additional sampling focused on representing the feather lice (Philopteridae) from a wider diversity of birds, as well as from each of the extant palaeognath orders: Apterygiformes (kiwis), Casuariiformes (cassowaries and emus), Rheiformes (rheas), Struthioniformes (ostriches), and Tinamiformes (tinamous).

### Extraction and whole-genome sequencing

Total genomic DNA from louse samples was extracted using Qiagen DNAeasy extraction kits with a modified protocol including a 48 h incubation step and elution from the filter with 52 µl of elution buffer. Total DNA was quantified with a Qubit 3.0 fluorometer and DNA was sonicated with a Covaris M220 to an average size of 300–400 nt. Paired-end libraries were prepared with a Kapa Library Preparation Kit (Kapa Biosystems). Libraries were pooled into equimolar concentration, quantified by qPCR and each pool was sequenced on one lane for 151–161 cycles on a HiSeq2500 (Illumina) using a TruSeq or HiSeq SBS sequencing rapid kit. Reads were 160 nt in length. All sequencing took place at the W.M. Keck Center at the University Illinois Urbana-Champaign. Adaptors were trimmed and Fastq files were created with Casava 1.8.2 or bcl2fastq v2.17.1.14 Conversion Software (Illumina).

### Gene assembly and orthology prediction

We used the automated Target Restricted Assembly Method (aTRAM v. 1.0)^22,23^ for three iterations to assemble 1107 orthologous gene sequences, using amino acid sequences from *Pediculus humanus*^24^ as a reference, the same gene set as in a previous study^4^. *Pediculus humanus* reference sequences were derived from a database of single copy orthologues from OrthoDB^25^. The contigs from the *best* file were then processed by Exonerate 2.2.0^26^ with the original *P. humanus* reference to identify exon boundaries and concatenate sequences^23^.

### Phylogenomic analyses

Orthologous coding gene sequences were translated with Geneious 11.1.5^27^ and aligned by amino acid sequence with PASTA 1.8.0^28^. Nucleotide alignments were then retrieved using a custom Python script based on the aligned amino acid data. Data were masked using a 40% gap threshold with trimAl 1.4^29^. Gene alignments with <50% of taxa sampled were excluded from analyses. In total 1075 alignments were analyzed. A final concatenated supermatrix was produced with Sequence Matrix 1.8^30^. Two nucleotide supermatrices were produced using Geneious for subsequent phylogenetic analyses: all sites included, and third codon positions excluded. Third positions showed considerable variation in base composition, so these alternative analyses accounted for this variation. The supermatrices were analyzed with PartitionFinder 2.1.1^31^ to identify an optimal partitioning scheme. The following parameters were set for all PartitionFinder analyses: branch lengths linked, GTR + G model, BIC model selection, rcluster search, and rcluster max set to 100.

Phylogenetic analyses using maximum likelihood methods were performed with ExaML 3.0.21^32^ and RAxML 8.2.11^33^. One hundred bootstrap replicates were completed in RAxML and the maximum likelihood hill-climbing algorithm was performed separately in ExaML. The hill-climbing algorithm was repeated four times using a parsimony starting tree and another four times using a random starting tree derived from RAxML to ensure that the topology with the highest likelihood was identified. Bootstrap replicates were then mapped onto the most favorable topology using SumTrees 4.1.0^34^. One hundred bootstrap replicates with the GTR + G model were implemented in RAxML. A gamma model was used in ExaML searches with the respective parsimony or random tree input. In total four analyses were performed with the described methods: all nucleotide sites (partitioned and unpartitioned) and third codon positions excluded (partitioned and unpartitioned).

A coalescent gene tree analysis was also performed using ASTRAL 5.5.9^35^. The analysis was performed on gene trees derived from single gene alignments of all nucleotide sites reconstructed with RAxML. Both bootstrapping (100 replicates) and the hill-climbing algorithm were performed for individual gene trees in RAxML using the GTR + G model. The concatenated bipartition files of all gene trees were used as the input for the ASTRAL analysis with default settings.

### Dating analysis

A molecular dating analysis was completed with MCMCTree^36^ in the PAML package under a relaxed clock. Dating analyses were performed on the topologies derived from the partitioned all nucleotide sites and third codon positions excluded maximum likelihood analyses. The following internal calibrations^4^ with soft bounds were used based on fossil evidence^37^ and evidence from codivergence events with their hosts: split between Nanopsocetae and Amphientometae (100 Mya minimum), between Menoponidae and its sister taxon (44 Mya minimum), codivergence of Old-World primates + Great Apes and their parasitic lice (20–25 Mya), and codivergence of Humans + Chimpanzees and their parasitic lice (5–7 Mya). The maximum root age was set to 200 Mya with soft bounds. The maximum root age was used to estimate the substitution rate across the entire phylogeny. These calibrations were also implemented in another dating analysis of parasitic lice^4^. Separate Monte Carlo Markov Chain runs were visualized for stationarity using the program Tracer 1.7.1^38^.

### Cophylogenomic analyses

Cophylogenetic analyses were performed with both louse phylogenies (all sites and 3rd sites removed) and two different host phylogenies based on Prum et al.^2^ and Jarvis et al.^1^ The Prum et al. tree was trimmed to include host taxa (family or order) parasitized by lice included in our study. The Jarvis et al. tree was also trimmed in this way, but missing lineages were added manually with relationships and divergence times based on Prum et al. In addition, the louse tree was pruned to include only feather lice (Philopteridae) of birds, excluding the lice of mammals and other groups of lice from birds. All four combinations of host-parasite trees were reconciled using Jane 4.0.1^7^. The bird and louse phylogenies were partitioned into three different time bins corresponding to the Cretaceous (>65 Mya), Palaeogene (65–23 Mya), and Neogene (23–2.5 Mya) Periods. Nodes from the four phylogenies were then assigned to each bin based on their divergence time estimates. Jane was then run on the four comparisons with Generations set to 100 and Population Size set to 500. The significance of the results was tested by randomizing the tip mapping 100 times, with 10 Generations and a Population Size of 50. Overall congruence between the bird and louse phylogenies was also tested using the distance-based methods Parafit^5^ and PACo^6^ using the R packages *ape* and *paco*, respectively. Both methods were run with 9999 permutations and the Cailliez correction for negative eigenvalues. PACo was run with the “r0” method (Supplementary Figs. 3 and 4).

### Reporting summary

Further information on research design is available in the Nature Research Reporting Summary linked to this article.

## Supplementary information

Supplementary Information

Reporting Summary

## Data Availability

Data generated during this study are available through the Illinois Databank (10.13012/B2IDB-0440388_V1)^39^. Raw read sequences are available in the NCBI SRA database (Supplementary Table 3).

## Code availability

All code and software used for this study has been cited and is available for public use.
