# Supplementary material for: Extensive host-switching of avian feather lice following the Cretaceous-Paleogene mass extinction event
Source: Commun Biol. 2019 Nov 29;2:445. doi: 10.1038/s42003-019-0689-7 (PMC6884534; doi:10.1038/s42003-019-0689-7)
Supplement: Supplementary file 1 — Supplementary Information [file 42003_2019_689_MOESM1_ESM.pdf]

Supplementary Figures

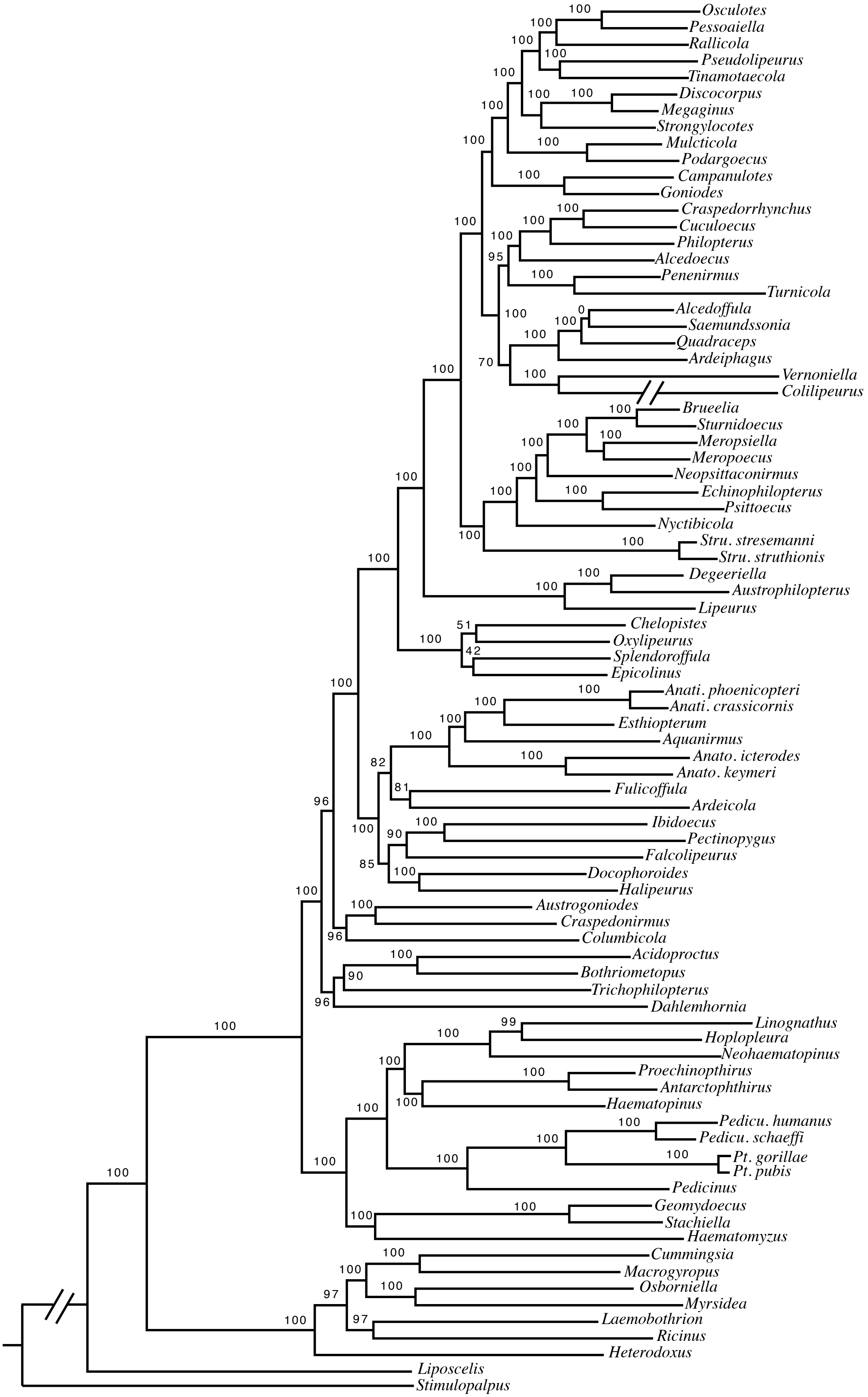
Osculotes
Pessoaiella
Rallicola
Pseudolipeurus
Tinamotaecola
Discocorpus
Megaginus
Strongylocotes
Mulcticola
Podargoecus
Campanulotes
Goniodes
Craspedorrhynchus
Cuculoecus
Philopterus
Alcedoecus
Penenirmus
Turnicola
Alcedoffula
Saemundssonia
Quadraceps
Ardeiphagus
Vernoniella
Colilipeurus
Brueelia
Sturnidoecus
Meropsiella
Meropoecus
Neopsittaconirmus
Echinophilopterus
Psittoecus
Nyctibicola
Stru. stresemanni
Stru. struthionis
Degeeriella
Austrophilopterus
Lipeurus
Chelopistes
Oxylipeurus
Splendoroffula
Epicolinus
Anati. phoenicopteri
Anati. crassicornis
Esthiopterum
Aquanirmus
Anato. icterodes
Anato. keymeri
Fulicoffula
Ardeicola
Ibidoecus
Pectinopygus
Falcolipeurus
Docophoroides
Halipeurus
Austrogoniodes
Craspedonirmus
Columbicola
Acidoproctus
Bothriometopus
Trichophilopterus
Dahlemhornia
Linognathus
Hoplopleura
Neohaematopinus
Proechinopthirus
Antarctophthirus
Haematopinus
Pedicu. humanus
Pedicu. schaeffi
Pt. gorillae
Pt. pubis
Pedicinus
Geomydoecus
Stachiella
Haematomyzus
Cummingsia
Macrogyropus
Osborniella
Myrsidea
Laemobothrion
Ricinus
Heterodoxus
Liposcelis
Stimulopalpus
100
100
100
100
100
100
100
100
100
100
100
100
100
95
100
100
0
100
100
70
100
100
100
100
100
100
100
100
100
100
100
100
100
51
42
100
100
100
100
100
82
81
100
90
100
85
100
96
100
96
100
90
96
100
100
99
100
100
100
100
100
100
100
100
100
100
100
100
100
100
97
97
100
100

Supplementary Figure 1: Phylogeny based on nonpartitioned maximum likelihood analysis of all codon positions. Nodes labelled with bootstrap support.

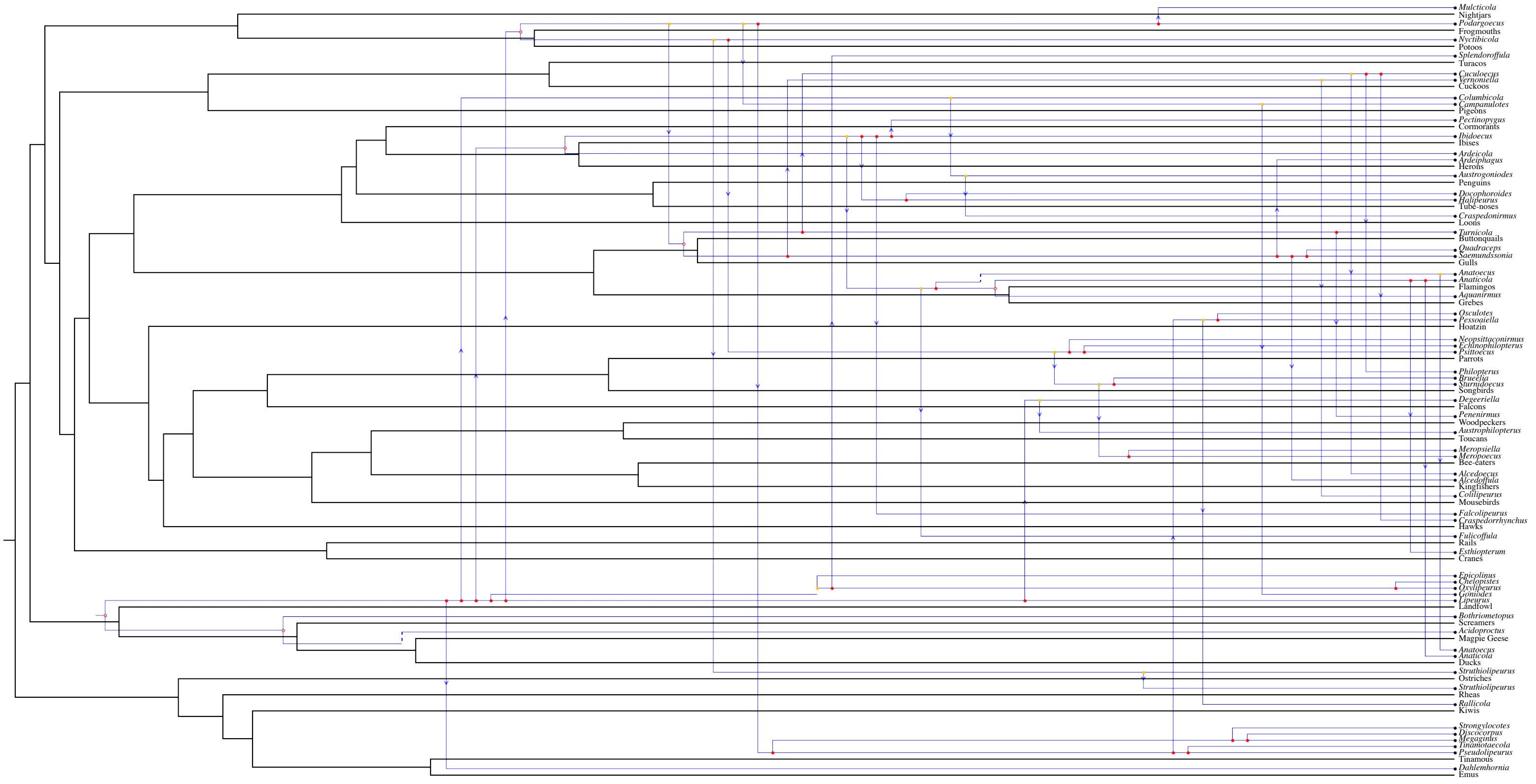
Mulcticola
Nightjars
Podargoecus
Frogmouths
Nyctibicola
Potoos
Splendoroffula
Turacos
Cuculoecus
Vernoniella
Cuckoos
Columbicola
Campanulotes
Pigeons
Pectinopygus
Cormorants
Ibidoecus
Ibises
Ardeicola
Ardeiphagus
Herons
Austrogoniodes
Penguins
Docophoroides
Halipeurus
Tube-noses
Craspedonirmus
Loons
Turnicola
Buttonquails
Quadraceps
Saemundssonia
Gulls
Anatoecus
Anaticola
Flamingos
Aquanirmus
Grebes
Osculotes
Pessoaiella
Hoatzin
Neopsittaconirmus
Echinophilopterus
Psittoecus
Parrots
Philopterus
Brueelia
Sturnidoecus
Songbirds
Degeeriella
Falcons
Penenirmus
Woodpeckers
Austrophilopterus
Toucans
Meropsiella
Meropoecus
Bee-eaters
Alcedoecus
Alcedoffula
Kingfishers
Colilipeurus
Mousebirds
Falcolipeurus
Craspedorrhynchus
Hawks
Fulicoffula
Rails
Esthiopterum
Cranes
Epicolinus
Chelopistes
Oxylipeurus
Goniodes
Lipeurus
Landfowl
Bothriometopus
Screamers
Acidoproctus
Magpie Geese
Anatoecus
Anaticola
Ducks
Struthiolipeurus
Ostriches
Struthiolipeurus
Rheas
Rallicola
Kiwis
Strongylocotes
Discocorpus
Megaginus
Tinamotaecola
Pseudolipeurus
Tinamous
Dahlemhornia
Emus

Supplementary Figure 2: One possible reconstruction from the Jane cophylogenetic analysis of the Prum *et al*.[2] topology (black) and feather louse topology (blue) derived from the partitioned maximum likelihood analysis with 3rd codon positions removed. Blue arrows indicate directionality of a host-switch. Hollow red circles identify cospeciation events. Filled colored (red and yellow) circles identify duplication events. Dashed lines represent a loss (sorting event).

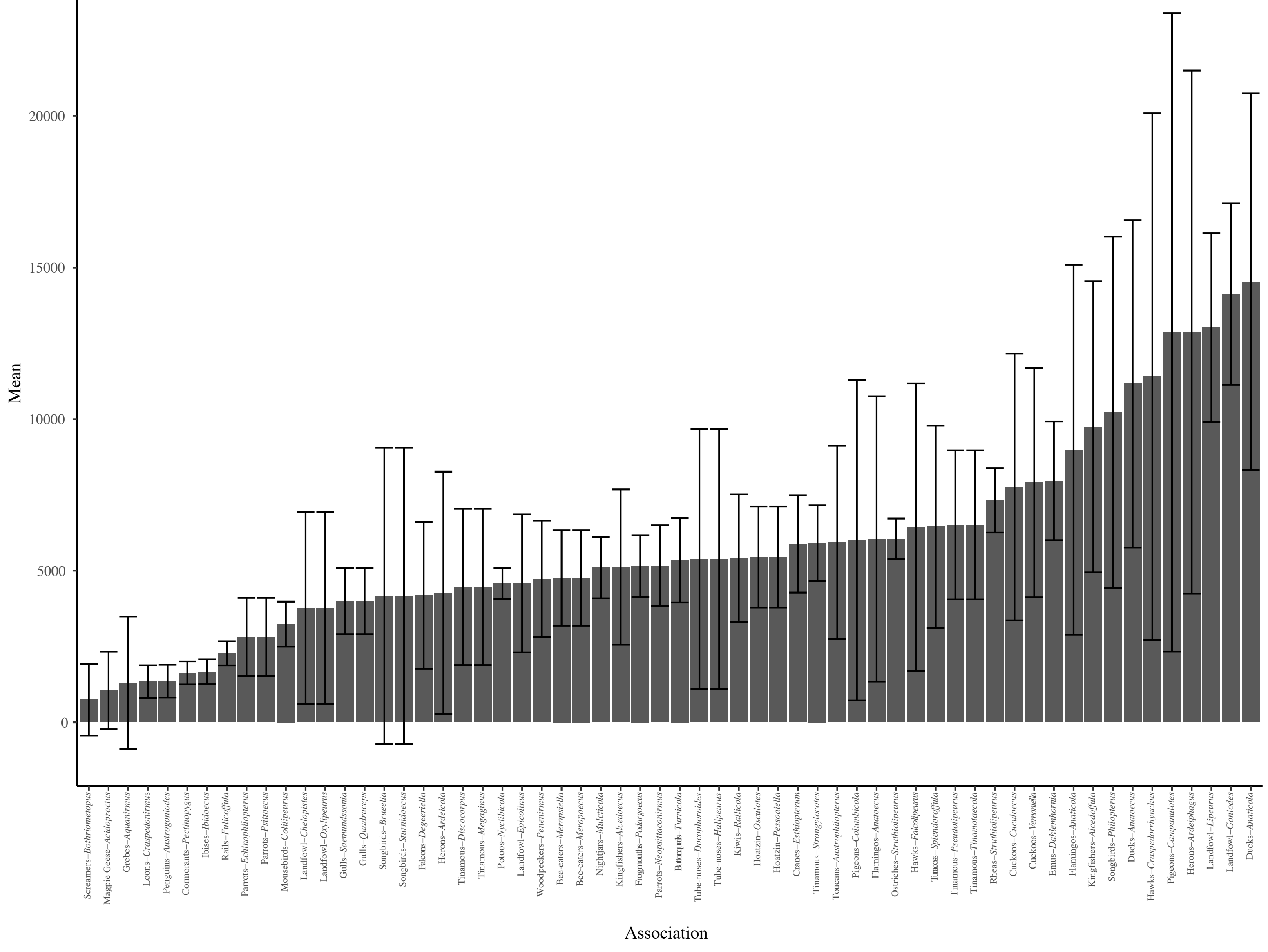
Mean
20000
15000
10000
5000
0
Screamers–*Bothriometopus*
Magpie Geese–*Acidoproctus*
Grebes–*Aquanirmus*
Loons–*Craspedonirmus*
Penguins–*Austrogoniodes*
Cormorants–*Pectinopygus*
Ibises–*Ibidoecus*
Rails–*Fulicoffula*
Parrots–*Echinophilopterus*
Parrots–*Psittoecus*
Mousebirds–*Colilipeurus*
Landfowl–*Chelopistes*
Landfowl–*Oxylipeurus*
Gulls–*Saemundssonia*
Gulls–*Quadraceps*
Songbirds–*Brueelia*
Songbirds–*Sturnidoecus*
Falcons–*Degeeriella*
Herons–*Ardeicola*
Tinamous–*Discocorpus*
Tinamous–*Megaginus*
Potoos–*Nyctibicola*
Landfowl–*Epicolinus*
Woodpeckers–*Penenirmus*
Bee-eaters–*Meropsiella*
Bee-eaters–*Meropoecus*
Nightjars–*Mulcticola*
Kingfishers–*Alcedoecus*
Frogmouths–*Podargoecus*
Parrots–*Neopsittaconirmus*
Buttonquails–*Turnicola*
Tube-noses–*Docophoroides*
Tube-noses–*Halipeurus*
Kiwis–*Rallicola*
Hoatzin–*Osculotes*
Hoatzin–*Pessoaiella*
Cranes–*Esthiopterum*
Tinamous–*Strongylocotes*
Toucans–*Austrophilopterus*
Pigeons–*Columbicola*
Flamingos–*Anatoecus*
Ostriches–*Struthiolipeurus*
Hawks–*Falcolipeurus*
Turacos–*Splendoroffula*
Tinamous–*Pseudolipeurus*
Tinamous–*Tinamotaecola*
Rheas–*Struthiolipeurus*
Cuckoos–*Cuculoecus*
Cuckoos–*Vernoniella*
Emus–*Dahlemhornia*
Flamingos–*Anaticola*
Kingfishers–*Alcedoffula*
Songbirds–*Philopterus*
Ducks–*Anatoecus*
Hawks–*Craspedorrhynchus*
Pigeons–*Campanulotes*
Herons–*Ardeiphagus*
Landfowl–*Lipeurus*
Landfowl–*Goniodes*
Ducks–*Anaticola*
Association

Supplementary Figure 3: Bar graph of the mean residual link scores between feather lice and their avian hosts as suggested by Paco analysis of the Prum *et al.*[2] topology and the feather louse maximum likelihood topology with 3rd positions removed. Whiskers and fences display the 95% confidence intervals produced that contains the true mean residual link score for each bird louse association. Statistics are derived from n = 9,999 permutations of the two topologies for respective bird louse associations tested.

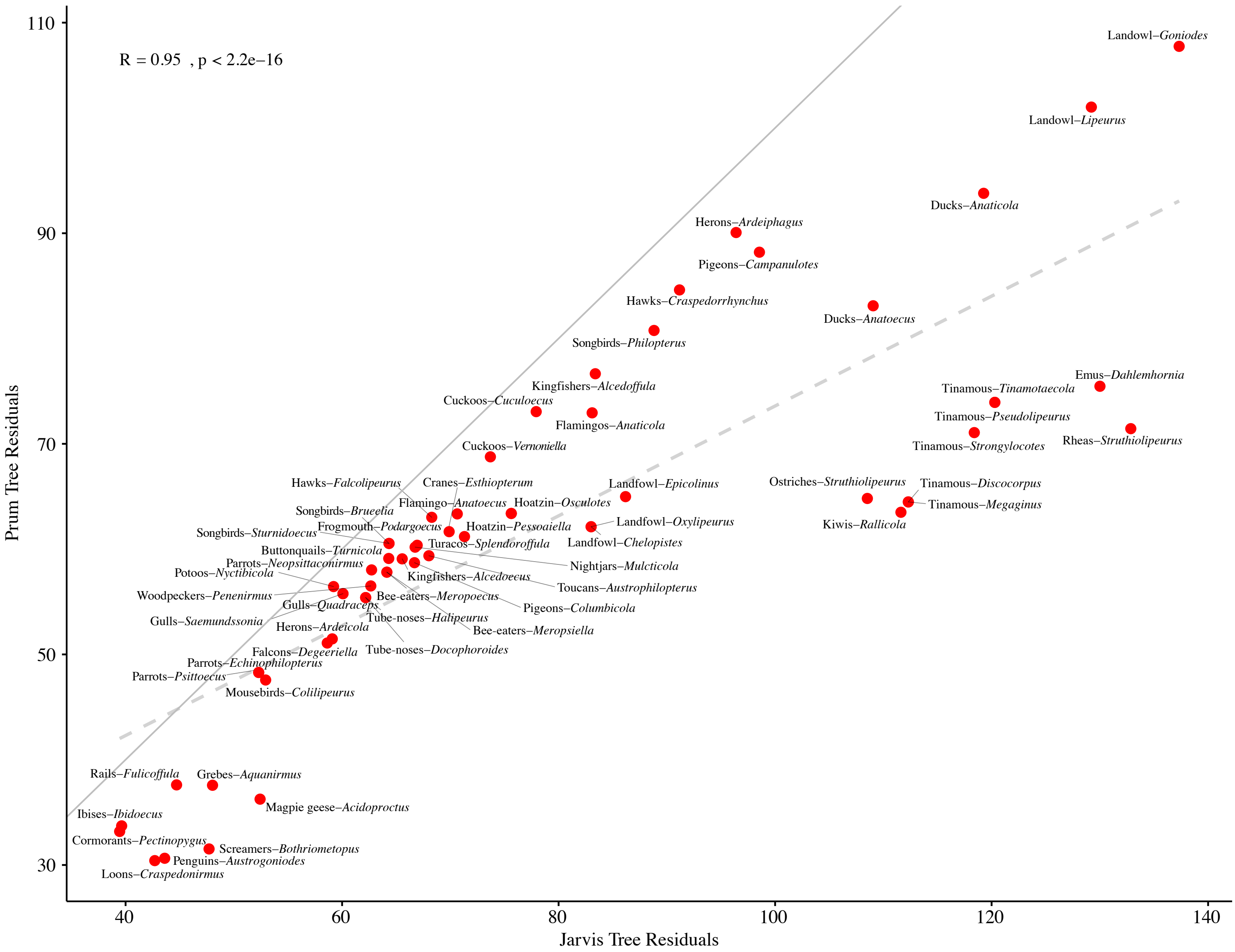

R = 0.95 , p < 2.2e−16
Prum Tree Residuals
Jarvis Tree Residuals
110
90
70
50
30
40
60
80
100
120
140
Landowl–Goniodes
Landowl–Lipeurus
Ducks–Anaticola
Herons–Ardeiphagus
Pigeons–Campanulotes
Hawks–Craspedorrhynchus
Ducks–Anatoecus
Songbirds–Philopterus
Kingfishers–Alcedoffula
Emus–Dahlemhornia
Tinamous–Tinamotaecola
Cuckoos–Cuculoecus
Tinamous–Pseudolipeurus
Flamingos–Anaticola
Cuckoos–Vernoniella
Tinamous–Strongylocotes
Rheas–Struthiolipeurus
Ostriches–Struthiolipeurus
Tinamous–Discocorpus
Hawks–Falcolipeurus
Cranes–Esthiopterum
Landfowl–Epicolinus
Tinamous–Megaginus
Songbirds–Brueelia
Flamingo–Anatoecus
Hoatzin–Osculotes
Landfowl–Oxylipeurus
Kiwis–Rallicola
Songbirds–Sturnidoecus
Frogmouth–Podargoecus
Hoatzin–Pessoaiella
Landfowl–Chelopistes
Turacos–Splendoroffula
Buttonquails–Turnicola
Parrots–Neopsittaconirmus
Nightjars–Mulcticola
Potoos–Nyctibicola
Kingfishers–Alcedoecus
Toucans–Austrophilopterus
Woodpeckers–Penenirmus
Bee-eaters–Meropoecus
Gulls–Quadraceps
Pigeons–Columbicola
Tube-noses–Halipeurus
Gulls–Saemundssonia
Herons–Ardeicola
Bee-eaters–Meropsiella
Falcons–Degeeriella
Tube-noses–Docophoroides
Parrots–Echinophilopterus
Parrots–Psittoecus
Mousebirds–Colilipeurus
Rails–Fulicoffula
Grebes–Aquanirmus
Magpie geese–Acidoproctus
Ibises–Ibidoecus
Cormorants–Pectinopygus
Screamers–Bothriometopus
Penguins–Austrogoniodes
Loons–Craspedonirmus

Supplementary Figure 4: Scatter plot of residuals from Paco analysis using the Prum *et al*.[2] versus Jarvis *et al*.[1] avian tree topologies when analyzed with the feather louse maximum likelihood tree topology with 3rd codon positions removed. Statistics are derived from n = 9,999 permutations of the two topologies for respective bird louse associations tested.

Supplementary Tables

Table 1:

Summary of Jane cophylogenetic analyses with Prum topology:

All nucleotide sites:

Solution ID	Cospeciations	Duplications	Duplications & Host Switches	Losses	Failures to Diverge	Cost	Ancestral Host	Host-switches to Palaeognaths	Ancestral host prior to Palaeognath host-switch
1	6	15	38	2	0	93	Galloanserae	4	Anseriformes, *Nyctibius* , *Podargus, Opisthocomus*
2	6	15	38	2	0	93	Galloanserae	4	Anseriformes, *Nyctibius, Podargus, Opisthocomus*
3	6	15	38	2	0	93	Galloanserae	4	Anseriformes, *Nyctibius, Podargus, Opisthocomus*
4	7	15	37	4	0	93	Galloanserae	4	Anseriformes, *Nyctibius, Podargus, Opisthocomus*
5	7	15	37	4	0	93	Galloanserae	4	Anseriformes, *Nyctibius, Podargus, Opisthocomus*
6	7	15	37	4	0	93	Galloanserae	4	Anseriformes, *Nyctibius, Podargus, Opisthocomus*
7	6	14	39	1	0	93	Galloanserae	4	Anseriformes, *Nyctibius, Podargus, Opisthocomus*
8	6	14	39	1	0	93	Galloanserae	4	Anseriformes, *Nyctibius, Podargus, Opisthocomus*
9	6	14	39	1	0	93	Galloanserae	4	Anseriformes, *Nyctibius, Podargus, Opisthocomus*
10	6	14	39	1	0	93	Galloanserae	4	Anseriformes, *Nyctibius, Podargus, Opisthocomus*
11	6	14	39	1	0	93	Galloanserae	4	Anseriformes, *Nyctibius, Podargus, Opisthocomus*
12	6	14	39	1	0	93	Galloanserae	4	Anseriformes, *Nyctibius, Podargus, Opisthocomus*
13	7	14	38	3	0	93	Galloanserae	4	Anseriformes, *Nyctibius, Podargus, Opisthocomus*
14	7	14	38	3	0	93	Galloanserae	4	Anseriformes, *Nyctibius, Podargus, Opisthocomus*
15	7	14	38	3	0	93	Galloanserae	4	Anseriformes, *Nyctibius, Podargus, Opisthocomus*
16	7	14	38	3	0	93	Galloanserae	4	Anseriformes, *Nyctibius, Podargus, Opisthocomus*
17	7	14	38	3	0	93	Galloanserae	4	Anseriformes, *Nyctibius, Podargus, Opisthocomus*
18	7	14	38	3	0	93	Galloanserae	4	Anseriformes, *Nyctibius, Podargus, Opisthocomus*
19	7	14	38	3	0	93	Galloanserae	4	Anseriformes, *Nyctibius, Podargus, Opisthocomus*
20	7	14	38	3	0	93	Galloanserae	4	Anseriformes, *Nyctibius, Podargus, Opisthocomus*
21	7	14	38	3	0	93	Galloanserae	4	Anseriformes, *Nyctibius, Podargus, Opisthocomus*

3rd codon positions removed:

Solution ID	Cospeciations	Duplications	Duplications & Host Switches	Losses	Failures to Diverge	Cost	Ancestral Host	Host-switches to Palaeognaths	Ancestral host prior to Palaeognath host-switch
22	6	15	38	2	0	93	Galloanserae	4	Galliformes, *Nyctibius, Podargus, Opisthocomus*
23	6	15	38	2	0	93	Galloanserae	4	Galliformes, *Nyctibius, Podargus, Opisthocomus*
24	6	15	38	2	0	93	Galloanserae	4	Galliformes, *Nyctibius, Podargus, Opisthocomus*
25	7	15	37	4	0	93	Galloanserae	4	Galliformes, *Nyctibius, Podargus, Opisthocomus*
26	7	15	37	4	0	93	Galloanserae	4	Galliformes, *Nyctibius, Podargus, Opisthocomus*
27	7	15	37	4	0	93	Galloanserae	4	Galliformes, *Nyctibius, Podargus, Opisthocomus*
28	6	14	39	1	0	93	Galloanserae	4	Galliformes, *Nyctibius, Podargus, Opisthocomus*
29	6	14	39	1	0	93	Galloanserae	4	Galliformes, *Nyctibius, Podargus, Opisthocomus*
30	6	14	39	1	0	93	Galloanserae	4	Galliformes, *Nyctibius, Podargus, Opisthocomus*
31	6	14	39	1	0	93	Galloanserae	4	Galliformes, *Nyctibius, Podargus, Opisthocomus*
32	6	14	39	1	0	93	Galloanserae	4	Galliformes, *Nyctibius, Podargus, Opisthocomus*
33	6	14	39	1	0	93	Galloanserae	4	Galliformes, *Nyctibius, Podargus, Opisthocomus*
34	6	14	39	1	0	93	Galloanserae	4	Galliformes, *Nyctibius, Podargus, Opisthocomus*
35	6	14	39	1	0	93	Galloanserae	4	Galliformes, *Nyctibius, Podargus, Opisthocomus*
36	6	14	39	1	0	93	Galloanserae	4	Galliformes, *Nyctibius, Podargus, Opisthocomus*
37	6	14	39	1	0	93	Galloanserae	4	Galliformes, *Nyctibius, Podargus, Opisthocomus*
38	6	14	39	1	0	93	Galloanserae	4	Galliformes, *Nyctibius, Podargus, Opisthocomus*
39	6	14	39	1	0	93	Galloanserae	4	Galliformes, *Nyctibius, Podargus, Opisthocomus*
40	7	14	38	3	0	93	Galloanserae	4	Galliformes, *Nyctibius, Podargus, Opisthocomus*
41	7	14	38	3	0	93	Galloanserae	4	Galliformes, *Nyctibius, Podargus, Opisthocomus*
42	7	14	38	3	0	93	Galloanserae	4	Galliformes, *Nyctibius, Podargus, Opisthocomus*
43	7	14	38	3	0	93	Galloanserae	4	Galliformes, *Nyctibius, Podargus, Opisthocomus*
44	7	14	38	3	0	93	Galloanserae	4	Galliformes, *Nyctibius, Podargus, Opisthocomus*
45	7	14	38	3	0	93	Galloanserae	4	Galliformes, *Nyctibius, Podargus, Opisthocomus*
46	7	14	38	3	0	93	Galloanserae	4	Galliformes, *Nyctibius, Podargus, Opisthocomus*
47	7	14	38	3	0	93	Galloanserae	4	Galliformes, *Nyctibius, Podargus, Opisthocomus*
48	7	14	38	3	0	93	Galloanserae	4	Galliformes, *Nyctibius, Podargus, Opisthocomus*
49	7	14	38	3	0	93	Galloanserae	4	Galliformes, *Nyctibius, Podargus, Opisthocomus*
50	7	14	38	3	0	93	Galloanserae	4	Galliformes, *Nyctibius, Podargus, Opisthocomus*
51	7	14	38	3	0	93	Galloanserae	4	Galliformes, *Nyctibius, Podargus, Opisthocomus*

Supplementary Table 1: Summary of Jane solutions derived from cophylogenetic analysis of the Prum avian tree topology and the respective feather louse topology analyzed (3rd codon positions removed and all nucleotide sites).

Table 2:
Summary of Jane cophylogenetic analsyses with Jarvis topology:

All nucleotide sites:

Solution ID	Cospeciations	Duplications	Duplications & Host Switches	Losses	Failures to Diverge	Cost	Ancestral Host	Host-switches to Palaeognaths	Ancestral host prior to Palaeognath host-switch
52	6	14	39	1	0	93	Galloanserae	3	Anseriformes, *Podargus* , *Nyctibius*
53	6	14	39	1	0	93	Galloanserae	3	Anseriformes, *Podargus* , *Nyctibius*
54	6	14	39	1	0	93	Galloanserae	3	Anseriformes, *Podargus* , *Nyctibius*
55	6	14	39	1	0	93	Galloanserae	3	Anseriformes, *Podargus* , *Nyctibius*
56	6	14	39	1	0	93	Galloanserae	3	Anseriformes, *Podargus* , *Nyctibius*
57	6	14	39	1	0	93	Galloanserae	3	Anseriformes, *Podargus* , *Nyctibius*
58	6	14	39	1	0	93	Galloanserae	3	Anseriformes, *Podargus* , *Nyctibius*
59	6	14	39	1	0	93	Galloanserae	3	Anseriformes, *Podargus* , *Nyctibius*
60	6	14	39	1	0	93	Galloanserae	3	Anseriformes, *Podargus* , *Nyctibius*
61	6	14	39	1	0	93	Galloanserae	3	Anseriformes, *Podargus* , *Nyctibius*
62	7	14	38	3	0	93	Galloanserae	3	Anseriformes, *Podargus* , *Nyctibius*
63	7	14	38	3	0	93	Galloanserae	3	Anseriformes, *Podargus* , *Nyctibius*
64	7	14	38	3	0	93	Galloanserae	3	Anseriformes, *Podargus* , *Nyctibius*
65	7	14	38	3	0	93	Galloanserae	3	Anseriformes, *Podargus* , *Nyctibius*
66	7	14	38	3	0	93	Galloanserae	3	Anseriformes, *Podargus* , *Nyctibius*
67	7	14	38	3	0	93	Galloanserae	3	Anseriformes, *Podargus* , *Nyctibius*
68	7	14	38	3	0	93	Galloanserae	3	Anseriformes, *Podargus* , *Nyctibius*
69	7	14	38	3	0	93	Galloanserae	3	Anseriformes, *Podargus* , *Nyctibius*
70	7	14	38	3	0	93	Galloanserae	3	Anseriformes, *Podargus* , *Nyctibius*
71	7	14	38	3	0	93	Galloanserae	3	Anseriformes, *Podargus* , *Nyctibius*
72	7	14	38	3	0	93	Galloanserae	3	Anseriformes, *Podargus* , *Nyctibius*
73	7	14	38	3	0	93	Galloanserae	3	Anseriformes, *Podargus* , *Nyctibius*
74	7	14	38	3	0	93	Galloanserae	3	Anseriformes, *Podargus* , *Nyctibius*
75	7	14	38	3	0	93	Galloanserae	3	Anseriformes, *Podargus* , *Nyctibius*
76	7	14	38	3	0	93	Galloanserae	3	Anseriformes, *Podargus* , *Nyctibius*
77	7	14	38	3	0	93	Galloanserae	3	Anseriformes, *Podargus* , *Nyctibius*
78	7	14	38	3	0	93	Galloanserae	3	Anseriformes, *Podargus* , *Nyctibius*
79	7	14	38	3	0	93	Galloanserae	3	Anseriformes, *Podargus* , *Nyctibius*
80	7	14	38	3	0	93	Galloanserae	3	Anseriformes, *Podargus* , *Nyctibius*
81	7	14	38	3	0	93	Galloanserae	3	Anseriformes, *Podargus* , *Nyctibius*
82	7	14	38	3	0	93	Galloanserae	3	Anseriformes, *Podargus* , *Nyctibius*
83	7	14	38	3	0	93	Galloanserae	3	Anseriformes, *Podargus* , *Nyctibius*
84	7	14	38	3	0	93	Galloanserae	3	Anseriformes, *Podargus* , *Nyctibius*
85	7	14	38	3	0	93	Galloanserae	3	Anseriformes, *Podargus* , *Nyctibius*

3rd codon positions removed:

Solution ID	Cospeciations	Duplications	Duplications & Host Switches	Losses	Failures to Diverge	Cost	Ancestral Host	Host-switches to Palaeognaths	Ancestral host prior to Palaeognath host-switch
86	6	14	39	1	0	93	Galloanserae	3	Galliformes, *Podargus* , *Nyctibius*
87	6	14	39	1	0	93	Galloanserae	3	Galliformes, *Podargus* , *Nyctibius*
88	6	14	39	1	0	93	Galloanserae	3	Galliformes, *Podargus* , *Nyctibius*
89	6	14	39	1	0	93	Galloanserae	3	Galliformes, *Podargus* , *Nyctibius*
90	6	14	39	1	0	93	Galloanserae	3	Galliformes, *Podargus* , *Nyctibius*
91	6	14	39	1	0	93	Galloanserae	3	Galliformes, *Podargus* , *Nyctibius*
92	6	14	39	1	0	93	Galloanserae	3	Galliformes, *Podargus* , *Nyctibius*
93	6	14	39	1	0	93	Galloanserae	3	Galliformes, *Podargus* , *Nyctibius*
94	6	14	39	1	0	93	Galloanserae	3	Galliformes, *Podargus* , *Nyctibius*
95	6	14	39	1	0	93	Galloanserae	3	Galliformes, *Podargus* , *Nyctibius*
96	6	14	39	1	0	93	Galloanserae	3	Galliformes, *Podargus* , *Nyctibius*
97	6	14	39	1	0	93	Galloanserae	3	Galliformes, *Podargus* , *Nyctibius*
98	6	14	39	1	0	93	Galloanserae	3	Galliformes, *Podargus* , *Nyctibius*
99	6	14	39	1	0	93	Galloanserae	3	Galliformes, *Podargus* , *Nyctibius*
100	6	14	39	1	0	93	Galloanserae	3	Galliformes, *Podargus* , *Nyctibius*
101	6	14	39	1	0	93	Galloanserae	3	Galliformes, *Podargus* , *Nyctibius*
102	7	14	38	3	0	93	Galloanserae	3	Galliformes, *Podargus* , *Nyctibius*
103	7	14	38	3	0	93	Galloanserae	3	Galliformes, *Podargus* , *Nyctibius*
104	7	14	38	3	0	93	Galloanserae	3	Galliformes, *Podargus* , *Nyctibius*
105	7	14	38	3	0	93	Galloanserae	3	Galliformes, *Podargus* , *Nyctibius*
106	7	14	38	3	0	93	Galloanserae	3	Galliformes, *Podargus* , *Nyctibius*
107	7	14	38	3	0	93	Galloanserae	3	Galliformes, *Podargus* , *Nyctibius*
108	7	14	38	3	0	93	Galloanserae	3	Galliformes, *Podargus* , *Nyctibius*
109	7	14	38	3	0	93	Galloanserae	3	Galliformes, *Podargus* , *Nyctibius*
110	7	14	38	3	0	93	Galloanserae	3	Galliformes, *Podargus* , *Nyctibius*
111	7	14	38	3	0	93	Galloanserae	3	Galliformes, *Podargus* , *Nyctibius*
112	7	14	38	3	0	93	Galloanserae	3	Galliformes, *Podargus* , *Nyctibius*
113	7	14	38	3	0	93	Galloanserae	3	Galliformes, *Podargus* , *Nyctibius*
114	7	14	38	3	0	93	Galloanserae	3	Galliformes, *Podargus* , *Nyctibius*
115	7	14	38	3	0	93	Galloanserae	3	Galliformes, *Podargus* , *Nyctibius*
116	7	14	38	3	0	93	Galloanserae	3	Galliformes, *Podargus* , *Nyctibius*
117	7	14	38	3	0	93	Galloanserae	3	Galliformes, *Podargus* , *Nyctibius*
118	7	14	38	3	0	93	Galloanserae	3	Galliformes, *Podargus* , *Nyctibius*
119	7	14	38	3	0	93	Galloanserae	3	Galliformes, *Podargus* , *Nyctibius*
120	7	14	38	3	0	93	Galloanserae	3	Galliformes, *Podargus* , *Nyctibius*
121	7	14	38	3	0	93	Galloanserae	3	Galliformes, *Podargus* , *Nyctibius*
122	7	14	38	3	0	93	Galloanserae	3	Galliformes, *Podargus* , *Nyctibius*
123	7	14	38	3	0	93	Galloanserae	3	Galliformes, *Podargus* , *Nyctibius*
124	7	14	38	3	0	93	Galloanserae	3	Galliformes, *Podargus* , *Nyctibius*
125	7	14	38	3	0	93	Galloanserae	3	Galliformes, *Podargus* , *Nyctibius*

Supplementary Table 2: Summary of Jane solutions derived from cophylogenetic analysis of the Jarvis avian tree topology and the respective feather louse topology analyzed (3rd codon positions removed and all nucleotide sites).

Louse species	Family	Host	Total Reads	SRR Accession
Trichophilopterus babakotophilus	Philopteridae	*Propithecus verreauxi*	38,735,400	SRR5308144
Dahlemhornia asymmetrica	Philopteridae	*Dromaius novaehollandiae*	65,382,868	SRR5308359
Struthiolipeurus stresemanni	Philopteridae	*Rhea americana*	106,684,732	SRR5308383
Struthiolipeurus struthionis	Philopteridae	*Struthio camelus*	66,838,096	SRR5308365
Rallicola sp.	Philopteridae	*Apteryx* sp.	54,822,042	SRR5308364
Strongylocotes lipogonus	Philopteridae	*Rhynchotus rufescens*	68,077,110	SRR5308142
Megaginus tataupensis	Philopteridae	*Crypturellus tataupa*	58,725,252	SRR5308131
Discocorpus mexicanus	Philopteridae	*Crypturellus cinnamomeus*	102,833,150	SRR5308387
Pseudolipeurus plumbeus	Philopteridae	*Crypturellus tataupa*	45,289,078	SRR5308356
Tinamotaecola elegans	Philopteridae	*Eudromia elegans*	91,157,284	SRR5308366
Bothriometopus macrocnemis	Philopteridae	*Chauna torquata*	125,502,194	SRR5088466
Acidoproctus rostratus	Philopteridae	*Dendrocygna viduata*	72,086,096	SRR5308389
Oxylipeurus chiniri	Philopteridae	*Ortalis vetula*	71,982,724	SRR5308134
Chelopistes texanus	Philopteridae	*Ortalis vetula*	61,406,098	SRR5308114
Epicolinus clavatus	Philopteridae	*Colinus virginianus*	73,404,240	SRR5308393
Goniodes ortygis	Philopteridae	*Colinus virginianus*	91,028,250	SRR5308120
Lipeurus caponis	Philopteridae	*Gallus gallus*	56,279,502	SRR5308373
Anatoecus icterodes	Philopteridae	*Anas cyanoptera*	63,288,740	SRR5308111
Anaticola crassicornis	Philopteridae	*Anas clypeata*	108,620,518	SRR5308340
Mulcticola bacurau	Philopteridae	*Nyctidromus albicollis*	63,680,908	SRR5308374
Podargoecus papuensis	Philopteridae	*Podargus papuensis*	38,463,532	SRR5308376
Nyctibicola longirostris	Philopteridae	*Nyctibius jamaicensis*	62,948,748	SRR5308388
Columbicola columbae	Philopteridae	*Columba livia*	56,608,482	SRR5308115
Campanulotes compar	Philopteridae	*Columba livia*	117,145,090	SRR5308113
Austrogoniodes waterstoni	Philopteridae	*Eudyptula minor*	84,049,742	SRR5308390
Craspedonirmus immer	Philopteridae	*Gavia immer*	54,000,574	SRR5308116
Pectinopygus varius	Philopteridae	*Phalacrocorax varius*	108,163,928	SRR5308135
Docophoroides brevis	Philopteridae	*Diomedea exulans*	54,250,984	SRR5308117
Halipeurus diversus	Philopteridae	*Puffinus tenuirostris*	52,500,588	SRR5308124
Quadraceps punctatus	Philopteridae	*Larus argentatus*	68,896,180	SRR5308139
Saemundssonia lari	Philopteridae	*Larus novaehollandiae*	38,719,424	SRR5308141
Turnicola sp.	Philopteridae	*Turnix pyrrothorax*	50,940,150	SRR5308379
Ibidoecus bisignatus	Philopteridae	*Plegadis chihi*	42,590,904	SRR5308126
Esthiopterum brevicephalum	Philopteridae	*Grus canadensis*	113,004,908	SRR5308385
Ardeiphagus cochlearius	Philopteridae	*Cochlearius cochlearius*	79,452,682	SRR5308384
Ardeicola expallidus	Philopteridae	*Bubulcus ibis*	75,255,050	SRR5308391
Fulicoffula longipila	Philopteridae	*Fulica americana*	52,765,362	SRR5308119
Aquanirmus occidentalis	Philopteridae	*Aechmophorus occidentalis*	92,913,330	SRR5308392
Anatoecus keymeri	Philopteridae	*Phoenicopterus chilensis*	98,579,854	SRR5308381
Anaticola phoenicopteri	Philopteridae	*Phoenicopterus chilensis*	88,679,252	SRR5308382
Alcedoecus sp.	Philopteridae	*Halcyon badia*	77,411,704	SRR5308110
Alcedoffula alcyonae	Philopteridae	*Ceryle alcyon*	41,932,010	SRR5308368
Osculotes curta	Philopteridae	*Opisthocomus hoazin*	81,287,340	SRR5308133
Pessoaiella absita	Philopteridae	*Opisthocomus hoazin*	81,580,082	SRR5308145
Splendoroffula sp.	Philopteridae	*Tauraco porphyrelopha*	51,425,552	SRR5308378
Austrophilopterus cancellosus	Philopteridae	*Ramphastos sulfuratus*	41,088,016	SRR5308369
Penenirmus auritus	Philopteridae	*Sphyrapicus varius*	58,531,158	SRR5308137
Cuculoecus africanus	Philopteridae	*Chrysococcyx cupreus*	62,937,116	SRR5308372
Vernoniella guimaraesi	Philopteridae	*Crotophaga ani*	46,644,520	SRR5308380
Colilipeurus obscurior	Philopteridae	*Colius colius*	19,267,276	SRR5308370
Falcolipeurus marginalis	Philopteridae	*Cathartes aura*	46,795,498	SRR5308118
Craspedorrhynchus subhaematopus	Philopteridae	*Accipiter cooperii*	95,915,920	SRR5308371
Meropoecus sp.	Philopteridae	*Merops ornatus*	65,254,844	SRR5308363
Meropsiella sp.	Philopteridae	*Merops ornatus*	37,474,984	SRR5308362
Degeeriella rufa	Philopteridae	*Falco berigora*	55,640,798	SRR5308223
Neopsittaconirmus bushae	Philopteridae	*Northiella haematogaster*	75,033,324	SRR5308361
Echinophilopterus claytoni	Philopteridae	*Northiella haematogaster*	61,015,492	SRR5308360
Psittoecus sp.	Philopteridae	*Cacatua galerita*	46,060,548	SRR5308377
Philopterus sp.	Philopteridae	*Tyrannus melancholicus*	53,357,072	SRR5308375
Brueelia antiqua	Philopteridae	*Catharus ustulatus*	140,698,976	SRR5308112
Sturnidoecus sp.	Philopteridae	*Lamprotornis purpureus*	59,263,926	SRR5308357
Geomydoecus aurei	Trichodectidae	*Thomomys bottae*	116,680,800	SRR5308121
Stachiella larseni	Trichodectidae	*Mustela vison*	38,023,782	SRR5308143
Haematomyzus elephantis	Haematomyzidae	*Elephas maximus*	94,800,010	SRR5308122
Haematopinus eurysternus	Haematopinidae	*Bos* sp.	64,236,000	SRR5308123
Linognathus spicatus	Linognathidae	*Connochaetes taurinus*	42,855,056	SRR5308129
Proechinopthirus fluctus	Echinophthiriidae	*Callorhinus ursinus*	42,855,056	SRR5308138
Antarctophthirus microchir	Echinophthiriidae	*Otaria flavescens*	104,403,830	SRR5088465
Hoplopleura arboricola	Hoplopleuridae	*Tamias amoenus*	174,420,882	SRR5088468
Neohaematopinus pacificus	Polyplacidae	*Tamias minimus*	154,952,566	SRR5088469
Pedicinus badius	Pedicinidae	*Procolobus rufoimtratus*	49,099,090	SRR5308136
Pthirus pubis	Pthiridae	*Homo sapiens*	91,120,738	SRR5088475
Pthirus gorillae	Pthiridae	*Gorilla beringei*	120,850,588	SRR5088474
Pediculus schaeffi	Pediculidae	*Pan troglodytes*	128,404,204	SRR1182279
Pediculus humanus	Pediculidae	*Homo sapiens*	147,704,686	SRR5088472
Ricinus sp.	Ricinidae	*Myiothlypis luteoviridis*	29,736,768	SRR5308140
Laemobothrion tinnunculi	Laemobothriidae	*Falco longipennis*	86,487,406	SRR5308127
Myrsidea sp.	Menoponidae	*Myiothlypis luteoviridis*	35,830,404	SRR5308132
Osborniella crotophagae	Menoponidae	*Crotophaga ani*	85,359,404	SRR5088470
Macrogyropus costalimai	Gyropidae	*Cuniculus paca*	65,940,856	SRR5308130
Cummingsia maculata	Trimenoponidae	*Lestoros inca*	66,405,676	SRR5308146
Heterodoxus spiniger	Boopidae	*Canis lupus*	84,240,242	SRR5308125
Liposcelis brunnea	Liposcelididae	Non-parasitic	74,000,000	SRR5308128
Stimulopalpus japonicus	Amphientomidae	Non-parasitic	148,541,936	SRR5088476

Supplementary Table 3: Summary of taxonomic sampling and host associations of individual feather lice collected. Number of reads produced from Illumina sequencing and SRR accession numbers are summarized.

References

1. Jarvis, E. D. *et al.* Whole-genome analyses resolve early branches in the tree of life of modern birds. *Science* **346**, 1320–1331 (2014).
2. Prum, R. O. *et al.* A comprehensive phylogeny of birds (Aves) using targeted next-generation DNA sequencing. *Nature* **526**, 569–573 (2015).
